# A genome-wide association study follow-up suggests a possible role for *PPARG* in systemic sclerosis susceptibility

**DOI:** 10.1186/ar4432

**Published:** 2014-01-09

**Authors:** Elena López-Isac, Lara Bossini-Castillo, Carmen P Simeon, María Victoria Egurbide, Juan José Alegre-Sancho, Jose Luis Callejas, José Andrés Roman-Ivorra, Mayka Freire, Lorenzo Beretta, Alessandro Santaniello, Paolo Airó, Claudio Lunardi, Nicolas Hunzelmann, Gabriela Riemekasten, Torsten Witte, Alexander Kreuter, Jörg H W Distler, Annemie J Schuerwegh, Madelon C Vonk, Alexandre E Voskuyl, Paul G Shiels, Jacob M van Laar, Carmen Fonseca, Christopher Denton, Ariane Herrick, Jane Worthington, Shervin Assassi, Bobby P Koeleman, Maureen D Mayes, Timothy RDJ Radstake, Javier Martin

**Affiliations:** 1 Instituto de Parasitología y Biomedicina López-Neyra, IPBLN-CSIC, Parque Tecnológico Ciencias de la Salud, Avenida del Conocimiento s/n 18016-Armilla, Granada, Spain; 2 Servicio de Medicina Interna, Hospital Valle de Hebron, Barcelona, Spain; 3 Department of Internal Medicine, Hospital Universitario Cruces, Barakaldo, Spain; 4 Department of Rheumatology, Hospital del Doctor Peset, Valencia, Spain; 5 Department of Internal Medicine, Hospital Clínico San Cecilio, Granada, Spain; 6 Rheumatology Department, Hospital Universitario La Fe, Valencia, Spain; 7 Department of Internal Medicine, Thrombosis and Vasculitis Unit, Complexo Hospitalario Universitario de Vigo, Vigo, Spain; 8 Referral Center for Systemic Autoimmune Diseases, Fondazione IRCCS Ca’ Granda Ospedale Maggioire Policlinico di Milano, Milan, Italy; 9 UO Reumatologia ed Immunologia Clinica, Spedali Civili, Brescia, Italy; 10 Department of Medicine, Università degli Studi di Verona, Verona, Italy; 11 Department of Dermatology, University of Cologne, Cologne, Germany; 12 Department of Rheumatology and Clinical Immunology, Charité University Hospital, and German Rheumatism Research Centre, a Leibniz Institute, Berlin, Germany; 13 Hannover Medical School, Hannover, Germany; 14 Ruhr University of Bochum, Bochum, Germany; 15 Department of Internal Medicine, Institute for Clinical Immunology, University of Erlangen-Nuremberg, Erlangen, Germany; 16 Department of Rheumatology, Leiden University Medical Center, Leiden, The Netherlands; 17 Department of Rheumatology, Radboud University Nijmegen Medical Center, Nijmegen, The Netherlands; 18 Department of Rheumatology, VU University Medical Center, Amsterdam, The Netherlands; 19 Section of Epigenetics, Inst. Cancer Sciences, MVLS, University of Glasgow, Glasgow, UK; 20 Institute of Cellular Medicine, Newcastle University, Newcastle, UK; 21 Centre for Rheumatology, Royal Free and University College Medical School, London, UK; 22 Arthritis Research UK Epidemiology Unit and NIHR Manchester Musculoskeletal Biomedical Research Unit, The University of Manchester, Manchester Academic Health Science Centre, Manchester, UK; 23 The University of Texas Health Science Center–Houston, Houston, TX, USA; 24 Section Complex Genetics, Department of Medical Genetics, University Medical Center Utrecht, Utrecht, The Netherlands; 25 Department of Rheumatology and Clinical Immunology, University Medical Center, Utrecht, The Netherlands

## Abstract

**Introduction:**

A recent genome-wide association study (GWAS) comprising a French cohort of systemic sclerosis (SSc) reported several non-HLA single-nucleotide polymorphisms (SNPs) showing a nominal association in the discovery phase. We aimed to identify previously overlooked susceptibility variants by using a follow-up strategy.

**Methods:**

Sixty-six non-HLA SNPs showing a *P* value <10^-4^ in the discovery phase of the French SSc GWAS were analyzed in the first step of this study, performing a meta-analysis that combined data from the two published SSc GWASs. A total of 2,921 SSc patients and 6,963 healthy controls were included in this first phase. Two SNPs, *PPARG* rs310746 and *CHRNA9* rs6832151, were selected for genotyping in the replication cohort (1,068 SSc patients and 6,762 healthy controls) based on the results of the first step. Genotyping was performed by using TaqMan SNP genotyping assays.

**Results:**

We observed nominal associations for both *PPARG* rs310746 (*P*_MH_ = 1.90 × 10^-6^, OR, 1.28) and *CHRNA9* rs6832151 (*P*_MH_ = 4.30 × 10^-6^, OR, 1.17) genetic variants with SSc in the first step of our study. In the replication phase, we observed a trend of association for *PPARG* rs310746 (*P* value = 0.066; OR, 1.17). The combined overall Mantel-Haenszel meta-analysis of all the cohorts included in the present study revealed that *PPARG* rs310746 remained associated with SSc with a nominal non-genome-wide significant *P* value (*P*_MH_ = 5.00 × 10^-7^; OR, 1.25). No evidence of association was observed for *CHRNA9* rs6832151 either in the replication phase or in the overall pooled analysis.

**Conclusion:**

Our results suggest a role of *PPARG* gene in the development of SSc.

## Introduction

Systemic sclerosis (SSc) is a complex autoimmune disease with heterogeneous clinical manifestations characterized by extensive fibrosis in the skin and multiple internal organs, vascular damage, and immune imbalance with autoantibody production [1]. SSc patients are commonly classified in two major subtypes: limited cutaneous SSc (lcSSc) and diffuse cutaneous SSc (dcSSc), the latter with more progressive fibrosis of the skin, lungs, and other internal organs and, ultimately, with worse prognosis [2].

The etiology of this disorder is still unclear. However, epidemiologic and genetic studies clearly reflect the existence of a complex genetic component together with the influence of environmental factors [1]. During recent years, great advances have been made in our knowledge of the genetic basis of SSc [3,4], in part, thanks to the two independent genome-wide associations studies (GWASs) conducted in Caucasian populations that have been recently published [5,6], and several consequent follow-up studies [7-10].

However, despite these advances, the number of currently known *loci* explaining the genetic component of SSc is limited. To date, 13 *loci* have been identified as genetic risk factors for SSc at the genome-wide significance level. In other autoimmune diseases with multifactorial inheritance, such as Crohn disease, ulcerative colitis, or systemic lupus erythematosus, individual GWAS scans and follow-up meta-analyses have identified more than 71, 47, and 35 susceptibility *loci*, respectively [11-13]. Therefore, it is expected that additional risk factors for SSc remain to be discovered, and further meta-analyses and large replication studies are needed to identify part of the missing heritability of this disease.

Follow-up studies focused on the so-called grey zone of the GWASs, where SNPs with tier 2 associations (*P* values between 5 × 10^-8^ and 5 × 10^-3^) are located, constitute one of the most useful GWAS data-mining methods, because possible real association signals could be masked in that area because of a lack of statistical power. On this basis, we aimed to perform a follow-up study of the SNPs located in the grey zone of the GWAS by Allanore *et al*. [6], taking advantage of our GWAS data sets. We hypothesize that using a larger cohort would increase the statistical power and might lead to the identification of new suitable SSc genetic risk factors.

## Methods

### Study design

In the first step of this study, we focused on the 90 GWAS-genotyped SNPs that reached a *P* value < 10^-4^ in the discovery phase of the GWAS carried out by Allanore *et al*. [6]. Then, we analyzed the SNPs overlapping with those included in Radstake *et al*. [5]. After excluding those SNPs located within *MHC* genes or in previously associated *loci*, data for 66 SNPs were selected. A meta-analysis including these 66 SNPs was performed on the combined data set from the two SSc GWASs, showing only two SNPs (rs310746 *PPARG* and rs6832151 *CHRNA9* genetic variants) with a *P* value < 10^-5^ (see later). These two genetic variants were genotyped in independent replication cohorts. Finally, we performed a meta-analysis for these two selected SNPs combining genotype data from both first and replication steps.

### Study population

The first step of the study comprised a total of 2,921 SSc patients and 6,963 healthy controls of Caucasian ancestry from two previously published GWASs (European, USA, and French) [5,6]. The replication cohort was composed of 1,068 SSc patients and 1,490 healthy controls from two case–control sets of European ancestry (Italy and United Kingdom). We also included 5,272 extra English controls from The Wellcome Trust Case Control Consortium for the replication step comprising a total of 6,762 controls for this stage.

All SSc patients fulfilled the classification criteria by LeRoy *et al*. [2]. Approval from the local ethical committees (Comité de Bioética del Consejo Superior de Investigaciones Científicas, U.O. Comitato di Etica e Sperimentazione Farmaci Fondazione IRCCS Ca’ Granda, Ospedale Maggiore Policlinico di Milano, Comitato Etico Azienda Ospedaliera Universitaria Integrata di Verona, The Ethics Committee of the Spedali Civili, Brescia, Royal Free Hospital and Medical School Research Ethics Committee, Manchester University Research Ethics Committee, Local Research Ethics Committee at Glasgow Royal Infirmary, Newcastle University Ethics Committee, Ethical Committee of the University Erlangen-Nuremberg, Local Ethics Committee of the Radboud University Nijmegen Medical Centre, Medical Ethics Review Committee of the VU University, Medische Ethische Commissie Leids Universitair Medisch Centrum, Ethics Review Board of the Ruhr University Bochum, Ethics Committee of the University of Cologne, Ethical Committee from the Charité University Hospital, Ethik-Kommission der MHH, Internal Review Board of Texas University), and written informed consents from all participants were obtained in accordance with the tenets of the Declaration of Helsinki.

### Genotyping

In the first stage, genotype data for the 66 selected SNPs were obtained from both published SSc GWASs [5,6]. QC filters and principal component analysis were applied to the GWASs data, as described in Radstake *et al*. [5] and Allanore *et al.*[6].

In the replication phase, DNA from patients and controls was obtained by using standard methods. Genotyping was performed by using TaqMan 5′ allele discrimination predesigned assays from Applied Biosystems (rs310746 ID: C___8756618_10; rs6832151 ID: C__29224385_10, Foster City, CA, USA) in both 7900HT Fast Real-Time PCR System (Applied Biosystems*, Foster City, C*A*, USA)*, and LightCycler 480 Real-Time PCR System (Roche Applied Science*,* Mannheim, Germany). Genotyping call rate was > 98% for both genotyped SNPs.

### Statistical analysis

Association analyses of the genotype data was carried out with StatsDirect V.2.6.6 (StatsDirect, Altrincham, UK) and PLINK V.1.07 [14] software. Statistical significance was calculated by 2 × 2 contingency tables and χ^2^ or Fisher Exact test, when necessary, to obtain *P* values, odds ratios (ORs), and 95% confidence intervals (CIs) in the population-specific analyses. Mantel-Haenszel tests under fixed effects or random effects, when appropriate, were performed to meta-analyze the combined data. Breslow–Day method (BD) was used to assess the homogeneity of the associations among the different populations (Breslow–Day *P* values <0.05 were considered statistically significant). Hardy-Weinberg equilibrium (HWE) was tested for all cohorts (HWE *P* values lower than 0.01 were considered to show significant deviation from the equilibrium). None of the included cohorts showed significant deviation from HWE for the two genotyped SNPs. Since the analyses were performed by using GWAS data, the statistical threshold for considering a *P* value as a significant *P* value in the allelic association analyses was set at 5 × 10^-8^.

The statistical power of the combined analysis was 70% for the *PPARG* rs310746 and 100% for the *CHRNA9* rs6832151 to detect associations with OR = 1.3 and a statistical significance of 5 × 10^-8^, according to Power Calculator for Genetic Studies 2006 software [15].

## Results

Table 1 shows the results of the 66 GWAS-genotyped SNPs selected for the combined meta-analysis of the two GWAS data sets performed in the first step of this study (see Additional file 1: Table S1 provides the results from both GWASs and the combined meta-analysis for the 66 selected SNPs). Two SNPs showed a *P* value lower than 10^-5^ (*PPARG* rs310746: *P*_MH_ = 1.90 × 10^-6^; OR, 1.28; CI, 95%, 1.12 to 1.47; and *CHRNA9* rs6832151: *P*_MH_ = 4.30 × 10^-6^, OR, 1.17; CI 95%, 1.08 to 1.27), and presented no significant Breslow-Day *P* values (*P*_BD_) showing homogeneity in the ORs among populations. Therefore, these two SNPs were selected to genotype in independent cohorts. Patients and healthy controls were found to be in HWE at 1% significance level for both selected SNPs.

**Table 1:** Meta-analysis of 66 GWAS-genotyped SNPs in scleroderma (SSc) patients and healthy controls of Caucasian origin

**Chr**	***Locus***	**SNP**	**Minor/major**	**MAF cases**	**MAF controls**	***P***_**MH**_	**OR (CI 95%)**^**a**^	***P***_**BD**_
3	*PPARG*	rs310746	C/T	0.108	0.086	1.90E-06	1.28 [1.12-1.47]	0.33
4	*CHRNA9 | RHOH*	rs6832151	G/T	0.315	0.281	4.30E-06	1.17 [1.075-1.27]	0.05
2	*DYSF*	rs11692280	A/G	0.195	0.220	2.31E-04	0.86 [0.80-0.93]	0.04
4	*PGDS*	rs17021463	T/G	0.393	0.421	2.45E-04	0.89 [0.83-0.94]	0.13
22	*DGCR6*	rs2543958	G/T	0.127	0.109	4.98E-04	1.18 [1.09-1.28]	0.09
17	*ORMDL3/GSDML*	rs8079416	C/T	0.435	0.461	1.13E-03	0.90 [0.85-0.94]	0.01
1	*-*	rs6679637	A/G	0.100	0.116	2.35E-03	0.85 [0.77-0.94]	0.03
1	*CSFR3*	rs4653210	G/T	0.111	0.122	3.71E-03	0.86 [0.78-0.95]	0.02
3	*PPARG/ TSEN2*	rs9855622	T/C	0.124	0.110	4.75E-03	1.14 [1.01-1.30]	2.94E-03
7	*CACNA2D1*	rs1544461	A/G	0.429	0.409	5.14E-03	1.09 [1.02-1.16]	7.81E-04
18	*CNDP2*	rs2241508	G/A	0.421	0.401	5.26E-03	1.09 [1.03-1.15]	2.94E-04
11	*PHF21A/CREB3L1*	rs7128538	A/G	0.491	0.470	5.51E-03	1.09 [1.02-1.16]	4.62E-03
14	*NPAS3*	rs1299512	G/A	0.228	0.211	7.76E-03	1.10 [1.00-1.21]	0.05
13	*RFC3*	rs7335534	G/A	0.398	0.415	8.61E-03	0.91 [0.84-0.99]	7.34E-04
8	*DDEF1*	rs7817803	A/C	0.437	0.421	0.012	1.08 [1.01-1.15]	2.01E-03
8	*DDE/*	rs3057	C/T	0.439	0.423	0.012	1.08 [1.01-1.15]	1.13E-03
17	*TMEM132E/CCDC16*	rs887081	T/G	0.115	0.129	0.013	0.88 [0.810.95]	5.33E-03
5	*CDH18*	rs1911856	T/C	0.059	0.048	0.013	1.18 [1.03-1.35]	3.24E-03
7	*CAV1*	rs2402091	A/G	0.110	0.122	0.014	0.88 [0.80-0.97]	0.02
7	*-*	rs1228966	A/G	0.222	0.208	0.015	1.09 [1.01-1.18]	2.29E-03
7	*SEMA3A*	rs1228870	T/G	0.222	0.208	0.017	1.09 [1.01-1.18]	3.15E-03
5	*LOC389293*	rs7708428	G/A	0.401	0.418	0.019	0.92 [0.87-0.98]	0.01
9	*XPA*	rs2808699	A/C	0.403	0.423	0.021	0.92 [0.87-0.98]	2.38E-04
8	*DDEF1*	rs7839523	G/T	0.440	0.425	0.021	1.07 [1.011-1.14]	1.10E-03
9	*XPA*	rs2805790	A/G	0.403	0.422	0.022	0.92 [0.87-0.98]	3.37E-04
3	*IRAK2*	rs11706450	T/C	0.465	0.482	0.024	0.93 [0.86-1.01]	9.00E-03
9	*XPA*	rs2805815	A/G	0.403	0.422	0.024	0.93 [0.87-0.99]	3.02E-04
2	*NOL10*	rs4668690	A/G	0.067	0.059	0.026	1.15 [1.01-1.30	3.10E-03
7	*-*	rs1029541	T/C	0.230	0.219	0.028	1.08 [1.01-1.17]	8.07E-04
9	*XPA*	rs2668797	A/G	0.071	0.112	0.029	0.933 [0.87-0.99]	2.48E-04
14	*-*	rs1036570	A/G	0.322	0.335	0.032	0.92 [0.85-1.014]	2.13E-03
3	*-*	rs4128236	T/C	0.322	0.306	0.034	1.07 [0.98-1.17]	3.87E-04
1	*-*	rs10925871	A/G	0.193	0.181	0.038	1.08 [1.00-1.17]	6.22E-03
7	*CADPS2*	rs2501439	G/A	0.418	0.432	0.042	0.93 [0.87-0.99]	7.49E-03
7	*-*	rs757747	T/C	0.229	0.218	0.047	1.07 [1.00-1.16]	1.29E-03
7	*WBSCR17*	rs4585627	T/C	0.323	0.308	0.051	1.07 [1.00-1.14]	4.70E-03
10	*-*	rs1254860	C/T	0.110	0.100	0.064	1.09 [0.99-1.21]	0.03
8	*DDEF1*	rs6470805	G/A	0.333	0.344	0.069	0.94 [0.88-1.00]	4.88E-03
9	*LCN9*	rs541131	G/A	0.400	0.385	0.071	1.06 [0.99-1.12]	1.78E-03
5	*CDH18*	rs2202798	T/C	0.080	0.069	0.078	1.11 [0.98-1.24]	9.17E-04
3	*TDGF1*	rs6799581	G/T	0.260	0.268	0.080	0.94 [0.86-1.03]	6.85E-04
5	*CDH18*	rs12655266	A/G	0.074	0.065	0.111	1.10 [0.98-1.24]	1.09E-03
4	*NPY2R*	rs2880417	G/A	0.292	0.281	0.117	1.05 [0.99-1.13]	2.07E-05
7	*-*	rs10272701	T/C	0.192	0.183	0.130	1.06 [0.98-1.15]	7.73E-03
8	*FBX032*	rs3739284	C/T	0.219	0.224	0.148	0.94 [0.87-1.01]	3.71E-03
6	*ASCC3*	rs7771570	C/T	0.492	0.482	0.153	1.04 [0.98-1.11]	2.97E-04
18	*PHLPP*	rs2877745	T/C	0.091	0.084	0.158	1.08 [0.98-1.18]	3.49E-03
21	*-*	rs2831511	T/C	0.393	0.403	0.175	0.95 [0.90-1.01]	1.54E-04
11	*OPCML*	rs10894623	T/G	0.291	0.281	0.175	1.04 [0.95-1.14]	8.98E-04
6	*ASCC3*	rs6919745	T/C	0.476	0.467	0.190	1.042 [0.98-1.11]	1.88E-04
4	*NPY2R*	rs13138293	G/T	0.308	0.299	0.195	1.05 [0.97-1.11]	8.20E-05
15	*SMAD3*	rs4147358	A/C	0.237	0.243	0.203	0.95 [0.86-1.04]	4.76E-04
17	*TMEM132E*	rs4795032	T/C	0.351	0.341	0.232	1.04 [0.95-1.13]	6.65E-04
9	*-*	rs10756265	A/G	0.342	0.352	0.251	0.96 [0.90-1.02]	2.18E-04
9	*-*	rs443042	G/A	0.366	0.376	0.261	0.96 [0.90-1.02]	2.01E-04
9	*SUSD3*	rs9696357	T/C	0.154	0.160	0.268	0.95 [0.87-1.03]	2.11E-03
12	*SFRS8*	rs10794423	C/T	0.439	0.442	0.282	0.96 [0.89-1.04]	1.22E-03
2	*ATP6V1C2*	rs7422405	A/G	0.428	0.433	0.385	0.97 [0.91-1.03]	1.93E-04
3	*RBMS3*	rs35883	A/G	0.457	0.4564	0.556	1.019 [0.94-1.10]	1.04E-04
21	*CHODL/ PRSS7*	rs2248200	C/T	0.484	0.4815	0.598	1.01 [0.96-1.07]	1.35E-03
21	*CHODL/PRSS7*	rs1688165	A/G	0.485	0.4820	0.628	1.01 [0.96-1.07]	8.62E-04
16	*ZNF423*	rs1477020	T/C	0.121	0.1235	0.649	0.97 [0.86-1.10]	8.30E-06
1	*C1QB*	rs631090	C/T	0.073	0.0702	0.729	1.02 [0.90-1.15]	3.73E-04
16	*ZNF423*	rs1990629	G/A	0.128	0.1308	0.736	0.98 [0.87-1.10]	2.13E-05
11	*OPCML*	rs11223273	T/C	0.275	0.2721	0.759	1.01 [0.92-1.10]	1.40E-04
19	*TSPAN16*	rs322151	T/C	0.252	0.2527	0.990	0.99 [0.94-1.06]	3.37E-04

N, 2,921 SSc/6,963 controls. ^a^Odds ratio for the minor allele. *Chr*, chromosome; *CI*, confidence interval; *MAF*, minor allele frequency; *OR*, odds ratio; *P*_BD_, Breslow–Day test *P* value; *P*_MH_, allelic Mantel-Haenszel fixed effects model *P* value; *SNP*, single nucleotide polymorphism.

In the replication phase, we observed a trend of association for the *PPARG* rs310746 genetic variant (*P* value = 0.066; OR = 1.17; CI 95%, 0.99 to 1.38) in the combined analysis of the two replication cohorts (Table 2, upper rows). However, no evidence of association was observed for *CHRNA9* rs6832151 either in the pooled analysis (Table 2, upper rows) or in the analysis of each individual population (see Additional file 2: Table S2).

**Table 2:** Analysis of rs310746 and rs6832151 minor allele frequencies in the GWASs, replication, and combined cohorts

**Cohort, *N *** **(cases/controls)**	**Chr**	***Locus***	**SNP**	**Minor/major**	**MAF cases**	**MAF controls**	***P***_**MH**_	**OR (CI 95%)a**	***P***_**BD**_
**GWASs**	3	*SYN2*|*PPARG*	rs310746	C/T	0.108	0.087	1.90E-06	1.28 [1.12-1.47]	0.334
2921/6963	4	*CHRNA9*|*RHOH*	rs6832151	G/T	0.315	0.282	4.30E-06	1.17 [1.075-1.27]	0.054
**Replication**	3	*SYN2*|*PPARG*	rs310746	C/T	0.099	0.103	0.066	1.17 [0.99-1.38]	0.231
1068/6762	4	*CHRNA9*|*RHOH*	rs6832151	G/T	0.296	0.280	0.962	0.99 [0.89-1.11]	0.934
**Combined**	3	*SYN2*|*PPARG*	rs310746	C/T	0.106	0.094	5.00E-07	1.25 [1.15-1.37]	0.324
3989/13725	4	*CHRNA9*|*RHOH*	rs6832151	G/T	0.310	0.281	1.07E-04^b^	1.12 [1.06-1.19]	0.017

^a^Odds ratio for the minor allele. ^b^*P* value from meta-analysis under random effects = 0.051; OR = 1.10 (0.99-1.22).

Chr, chromosome; CI, confidence interval; MAF, minor allele frequency; OR, odds ratio; P_BD_, Breslow–Day test *P* value; P_MH_, allelic Mantel-Haenszel fixed-effects model P value; SNP, single-nucleotide polymorphism.

**Figure 1:**
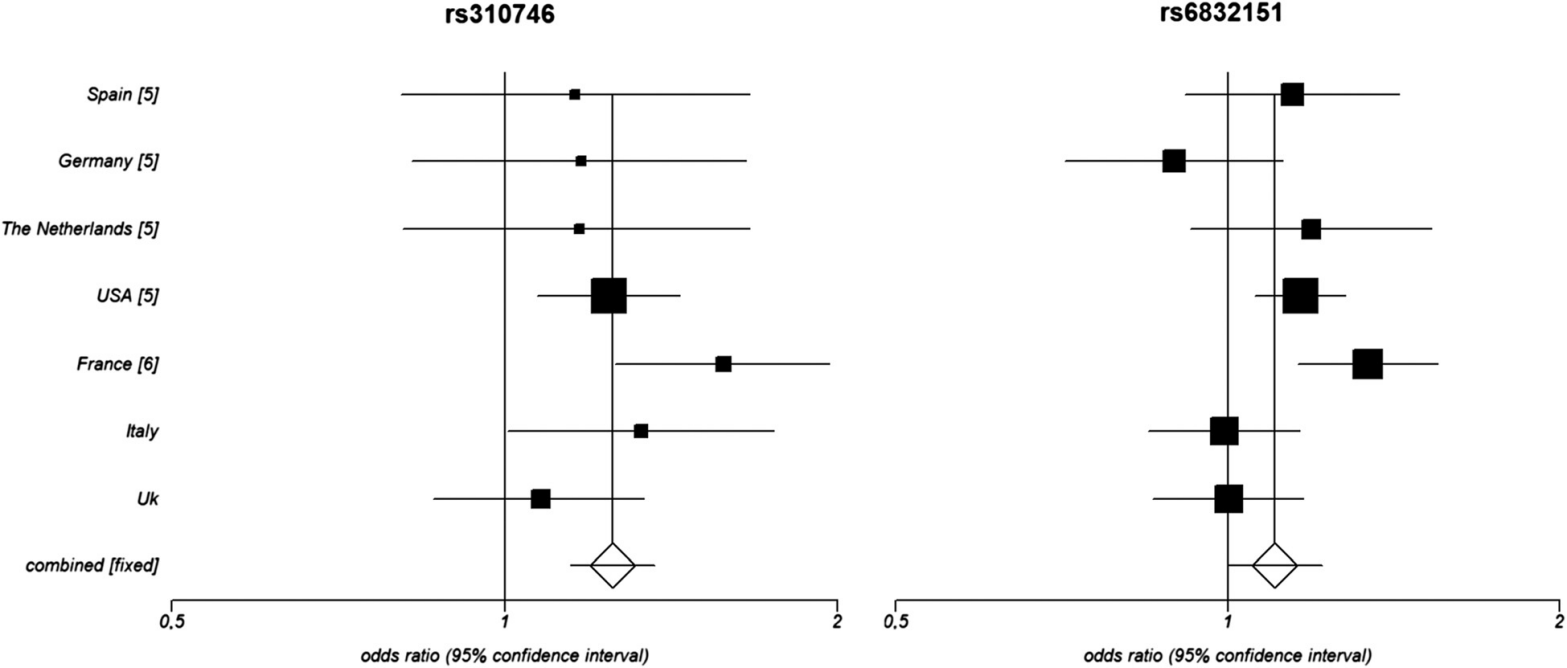
**Forest plots of *PPARG *rs310746 and *CHRNA9 *rs6832151.** Forest plots showing the odds ratios and confidence intervals of both *PPARG* rs310746 (under a fixed-effects model) and *CHRNA9* rs6832151 (under a random-effects model) in all the populations included in the combined analysis.

Finally, we combined the results from both steps of the study and performed a Mantel-Haenszel meta-analysis observing that the *PPARG* genetic variant showed suggestive evidence of association with SSc (*P*_MH_ = 5.00 × 10^-7^; OR = 1.25; CI, 95%, 1.15 to 1.37) (Table 2, lower rows; Figure 1). However, *CHRNA9* rs6832151 showed no evidence of association with the disease when the meta-analysis was performed either under a random-effects model (heterogeneity of the ORs was observed for this SNP; *P* value = 5.10 × 10^-2^, OR = 1.10; CI 95%, 0.99 to 1.22), or a fixed-effects model (*P* value = 1.07 × 10^-4^; OR = 1.12; CI 95%, 1.06 to 1.19) (Table 2, lower rows; Figure 1).

## Discussion

In this study we conducted a meta-analysis combining previously published SSc GWASs data for 66 SNPs and analyzed the possible role of two selected SNPs, *PPARG* rs310746 and *CHRNA9* rs6832151, in SSc risk by using independent replication cohorts.

Meta-analyses are a useful tool to increase the statistical power of genetic studies, thus improving the accuracy of the estimations of statistical significance. Of note, associations identified from a single GWAS often tend to have inflated effect sizes [16]. On this basis, our data suggest that most signals from the grey zone observed in the discovery phase of the GWAS by Allanore *et al*. [6] presented inflated effect sizes, also called the winner’s curse. In fact, this effect was already observed in the replication study conducted by our group for the novel SSc genetic risk factors identified by Allanore *et al*. [6], in which we could not replicate the association described for *RHOB*[17].

Our overall combined meta-analysis showed that the association of the *PPARG* rs310746 genetic variant with SSc remained with a nominal but non-genome-wide significant *P* value. This SNP is located upstream of *PPARG*, which encodes the peroxisome proliferator-activated receptor gamma (PPARG). *PPARG* was initially identified in adipose tissue, where this nuclear receptor plays important roles in adipogenesis, insulin sensitivity, and homeostasis [18]. Interestingly, during recent years, several studies have identified a novel role of PPARG as an antifibrotic effector. Thus, it has been reported that fibroblasts exposure to pharmacologic PPARG ligands give rise to suppression of collagen synthesis, myofibroblast differentiation, and other TGF-β-induced fibrotic responses *in vitro*[19-21]. Moreover, functional studies showed that PPARG agonist attenuated dermal fibrosis in mice with bleomycin-induced scleroderma [22,23].

These findings are remarkable in SSc, in which fibrosis is one of the main hallmarks of the disease. In this regard, Wei *et al*. [24] demonstrated that PPARG expression and function are impaired in SSc patients. Therefore, defects in *PPARG* expression may influence the uncontrolled progression of fibrosis in SSc. In addition, *PPARG* has been associated with other autoimmune diseases, such as inflammatory bowel disease [25,26] and psoriatic arthritis [27], and it is also a confirmed susceptibility *locus* in type 2 diabetes mellitus [28].

Although *PPARG* was the most likely biologic candidate gene for the reported suggestive association signal, we could not rule out *TIMP4* as another possible gene for this signal. Further analyses are required to elucidate the functional implication of the reported signal.

Regarding the *CHRNA9* genetic variant, despite the suggestive association found in the first step of the present study, the overall combined meta-analysis did not show evidence of association with SSc. Moreover, the effect size of the analyzed genetic variant was heterogeneous between the different populations. Although our data showed heterogeneity and lack of association in this *locus*, a slight or modest effect of *CHRNA9* cannot be ruled out, and further studies will be required to determine whether this region is associated with SSc.

It is worth mentioning that the analyzed *CHRNA9* SNP has been previously associated with Graves disease (first, through a GWAS performed in the Chinese Han population [29], and subsequently, in a replication study performed in a Polish Caucasian population [30]), but this is the only reported association between this gene and an autoimmune disease.

## Conclusion

In conclusion, we report a suggestive association between *PPARG* rs310746 and SSc. However, further studies are needed to establish this *locus* firmly as a new susceptibility SSc genetic risk factor.

## Abbreviations

BD test: Breslow-day test; CHRNA9: cholinergic receptor nicotinic, Alpha 9; CI: Confidence interval; dcSSc: diffuse cutaneous systemic sclerosis; DNA: deoxyribonucleic acid; GWAS: genome-wide association study; HLA: Human leukocyte antigen; HWE: Hardy-Weinberg equilibrium; lcSSc: limited cutaneous systemic sclerosis; MAF: minor allele frequency; MHC: major histocompatibility complex; OR: Odds ratio; PCR: polymerase chain reaction; PPARG: peroxisome proliferator-activated receptor gamma; SNP: single nucleotide polymorphism; SSc: systemic sclerosis; TGF: transforming growth factor.

## Competing interests

The authors declare that they have no competing interests.

## Authors’ contributions

ELI and LBC contributed to the analysis and interpretation of data and the drafting the manuscript. CPS and AH participated in the acquisition of data and the drafting of the manuscript. JM contributed to the conception and design of the study and critically revised the manuscript. MVE, JJAS, JLC, JARI, MF, LB, AS, PA, CL, NH, GR, TW, AK, JHWD, AJS, MCV, AEV, PGS, JMvL, CF, CD, JW, SA, BPK, MDM, TRDJR, and the Spanish Scleroderma Group were involved in the acquisition of data and the revision of the manuscript. All authors read and approved the final manuscript.

## Supplementary Material

Additional file 1 **GWASs results from Allanore *et al*. [**[6]**] and Radstake *et al*. [**[5]**], and combined meta-analysis.** Description: this file contains Additional file 1: Table S1 showing the results for the 66 selected SNPs in Allanore *et al*. and Radstake *et al*. GWASs, followed by the results of the combined meta-analysis performed in the present study. Click here for file

Additional file 2 **Genotype and minor allele frequencies of rs310746 and rs6832151 SNPs in two European cohorts (Replication-step).** Description: this file contains: Additional file 2: Table S2 showing the genotype and allele distributions of rs310746 and rs6832151 genetic variants in two European cohorts (1032 SSc cases and 6700 controls). Click here for file

## Spanish Scleroderma Group

Spanish Scleroderma Group: Norberto Ortego-Centeno and Raquel Ríos, Unidad de Enfermedades Sistémicas Autoinmunes, Department of Internal Medicine, Hospital Clínico Universitario San Cecilio, Granada; Nuria Navarrete, Department of Internal Medicine, Hospital Virgen de las Nieves, Granada; Rosa García Portales, Department of Rheumatology, Hospital Virgen de la Victoria, Málaga; María Teresa Camps, Department of Internal Medicine, Hospital Carlos Haya, Málaga; Antonio Fernández-Nebro, Department of Rheumatology, Hospital Carlos Haya, Málaga; María F. González-Escribano, Department of Immunology, Hospital Virgen del Rocío, Sevilla; Julio Sánchez-Román, Francisco José García-Hernández and Mª Jesús Castillo, Department of Internal Medicine, Hospital Virgen del Rocío, Sevilla; Mª Ángeles Aguirre and Inmaculada Gómez-Gracia, Department of Rheumatology, Hospital Reina Sofía/IMIBIC, Córdoba; Patricia Carreira, Department of Rheumatology. Hospital Universitario 12 de Octubre, Madrid; Benjamín Fernández-Gutiérrez and Luis Rodríguez-Rodríguez, Department of Rheumatology, Hospital Clínico San Carlos, Madrid; Esther Vicente, Department of Rheumatology, Hospital La Princesa, Madrid; José Luis Andreu and Mónica Fernández de Castro, Department of Rheumatology, Hospital Puerta de Hierro Majadahonda, Madrid; Paloma García de la Peña, Department of Rheumatology, Hospital Madrid Norte Sanchinarro, Madrid; Francisco Javier López-Longo and Lina Martínez, Department of Rheumatology, Hospital General Universitario Gregorio Marañón, Madrid; Vicente Fonollosa and Carmen Pilar Simeón, Department of Internal Medicine, Hospital Valle de Hebrón, Barcelona; Gerard Espinosa, Department of Internal Medicine, Hospital Clinic, Barcelona; Iván Castellví, Department of Rheumatology, Hospital de la Santa Creu i Sant Pau, Barcelona; Carlos Tolosa, Department of Internal Medicine, Hospital Parc Tauli, Sabadell; Anna Pros, Department of Rheumatology, Hospital Del Mar, Barcelona; Mónica Rodríguez Carballeira, Department of Internal Medicine, Hospital Universitari Mútua Terrasa, Barcelona; Francisco Javier Narváez, Department of Rheumatology, Hospital Universitari de Bellvitge, Barcelona; Manel Rubio Rivas, Department of Internal Medicine, Hospital Universitari de Bellvitge, Barcelona; Vera Ortiz Santamaría, Department of Rheumatology, Hospital General de Granollers, Granollers; Miguel Ángel González-Gay, Department of Rheumatology, Hospital Universitario Marqués de Valdecilla, IFIMAV, Santander; Bernardino Díaz and Luis Trapiella, Department of Internal Medicine, Hospital Central de Asturias, Oviedo; Adrián Sousa, Unidad de Trombosis y Vasculitis, Department of Internal Medicine, Hospital Xeral-Complexo Hospitalario Universitario de Vigo, Vigo; Patricia Fanlo Mateo, Department of Internal Medicine Hospital Virgen del Camino, Pamplona; Luis Sáez-Comet, Unidad de Enfermedades Autoinmunes Sistémicas, Department of Internal Medicine, Hospital Universitario Miguel Servet, Zaragoza; Federico Díaz and Vanesa Hernández, Department of Rheumatology, Hospital Universitario de Canarias, Tenerife; Emma Beltrán, Department of Rheumatology, Hospital General Universitario de Valencia, Valencia; and Francisco J. Blanco García and Natividad Oreiro, Department of Rheumatology, INIBIC-Hospital Universitario A Coruña, La Coruña.
