# Supplementary material for: A genome-wide association study follow-up suggests a possible role for PPARG in systemic sclerosis susceptibility
Source: Arthritis Res Ther. 2014 Jan 9;16(1):R6. doi: 10.1186/ar4432 (PMC3978735; doi:10.1186/ar4432)
Supplement: Additional file 1 — GWASs results from Allanore et al. [[6]] and Radstake et al. [[5]], and combined meta-analysis. Description: this file contains Additional file 1: Table S1 showing the results for the 66 selected SNPs in Allanore et al. and Radstake et al. GWASs, followed by the results of the combined meta-analysis performed in the present study. [file ar4432-S1.docx]

**Additional file 1: Table S1. GWASs results from Allanore *et al*. and Radstake *et al*., and combined meta-analysis.**

|  |  |  | **Allanore *et al*.** | | | **Radstake *et al*.** | | | | **Mantel-Haenszel Meta-analysis** | | | |
| --- | --- | --- | --- | --- | --- | --- | --- | --- | --- | --- | --- | --- | --- |
| **Chr** | ***Locus*** | **SNP** | **MAF Cases/ Controls** | ***P*_value_** | **OR*** | **MAF Cases/ Controls** | ***P*_value_** | **OR*** | ***P*_BD_** | **MAF Cases/ Controls** | ***P*_MH_** | **OR [CI 95%]*** | ***P*_BD_** |
| 3 | *PPARG* | rs310746 | 0.121/ 0.08 | 6.15E-05 | 1.55 | 0.104/0.088 | 1.11E-03 | 1.21 | 0.970 | 0.108/0.086 | 1.90E-06 | 1.28 [1.12-1.47] | 0.33 |
| 4 | *CHRNA9 \| RHOH* | rs6832151 | 0.347/ 0.284 | 6.11E-05 | 1.34 | 0.307/0.280 | 2.02E-03 | 1.12 | 0.168 | 0.315/0.281 | 4.30E-06 | 1.17 [1.075-1.27] | 0.05 |
| 2 | *DYSF* | rs11692280 | 0.166/ 0.228 | 1.08E-05 | 0.67 | 0.201/0.217 | 0.059 | 0.92 | 0.974 | 0.195/0.22 | 2.31E-04 | 0.86 [0.80-0.93] | 0.04 |
| 4 | *PGDS* | rs17021463 | 0.384/ 0.451 | 9.48E-05 | 0.76 | 0.395/0.410 | 0.035 | 0.92 | 0.918 | 0.393/0.421 | 2.45E-04 | 0.89 [0.83-0.94] | 0.13 |
| 22 | *DGCR6* | rs2543958 | 0.149/ 0.105 | 7.46E-05 | 1.49 | 0.122/0.110 | 0.060 | 1.1 | 0.703 | 0.127/0.109 | 4.98E-04 | 1.18 [1.09-1.28] | 0.09 |
| 17 | *ORMDL3 \| GSDML* | rs8079416 | 0.406/ 0.48 | 1.11E-05 | 0.73 | 0.443/0.454 | 0.159 | 0.95 | 0.430 | 0.435/0.461 | 1.13E-03 | 0.90 [0.85-0.94] | 0.01 |
| 1 | *-* | rs6679637 | 0.081/ 0.125 | 6.29E-05 | 0.62 | 0.105/0.113 | 0.190 | 0.92 | 0.728 | 0.1/0.116 | 2.35E-03 | 0.85 [0.77-0.94] | 0.03 |
| 1 | *CSFR3* | rs4653210 | 0.098/ 0.147 | 2.60E-05 | 0.63 | 0.114/0.114 | 0.273 | 0.94 | 0.770 | 0.111/0.122 | 3.71E-03 | 0.86 [0.78-0.95] | 0.02 |
| 3 | *TSEN2* | rs9855622 | 0.145/ 0.096 | 1.64E-06 | 1.66 | 0.119/0.114 | 0.389 | 1.04 | 0.451 | 0.124/0.11 | 4.75E-03 | 1.14 [1.01-1.30] | 2.94E-03 |
| 7 | *CACNA2D1* | rs1544461 | 0.45/ 0.38 | 3.64E-05 | 1.33 | 0.424/0.419 | 0.318 | 1.03 | 0.038 | 0.429/0.409 | 5.14E-03 | 1.09 [ 1.02-1.16] | 7.81E-04 |
| 18 | *CNDP2* | rs2241508 | 0.457/ 0.386 | 1.80E-05 | 1.35 | 0.413/0.406 | 0.338 | 1.03 | 0.012 | 0.421/0.401 | 5.26E-03 | 1.09 [1.03-1.15] | 2.94E-04 |
| 11 | *PHF21A \| CREB3L1* | rs7128538 | 0.509/ 0.436 | 2.55E-05 | 1.33 | 0.486/0.485 | 0.525 | 1.02 | 0.245 | 0.491/0.47 | 5.51E-03 | 1.09 [1.02-1.16] | 4.62E-03 |
| 14 | *NPAS3* | rs1299512 | 0.259/ 0.203 | 8.34E-05 | 1.37 | 0.221/0.214 | 0.346 | 1.04 | 0.949 | 0.228/0.211 | 7.76E-03 | 1.10 [1.00-1.21] | 0.05 |
| 13 | *RFC3* | rs7335534 | 0.38/ 0.448 | 9.70E-05 | 0.76 | 0.403/0.404 | 0.359 | 0.96 | 0.032 | 0.398/0.415 | 8.61E-03 | 0.91 [0.84-0.99] | 7.34E-04 |
| 8 | *DDEF1* | rs3057 | 0.47/ 0.397 | 2.09E-05 | 1.34 | 0.432/0.432 | 0.547 | 1.02 | 0.134 | 0.439/0.423 | 0.012 | 1.08 [1.01-1.15] | 1.13E-03 |
| 8 | *DDEF1* | rs7817803 | 0.464/ 0.394 | 4.73E-05 | 1.32 | 0.431/0.430 | 0.470 | 1.02 | 0.126 | 0.437/0.421 | 0.012 | 1.08 [1.01-1.15] | 2.01E-03 |
| 5 | *CDH18* | rs1911856 | 0.075/ 0.042 | 1.04E-05 | 1.88 | 0.055/0.051 | 0.576 | 1.04 | 0.406 | 0.059/0.048 | 0.013 | 1.18 [1.03-1.35] | 3.24E-03 |
| 17 | *TMEM132E \| CCDC16* | rs887081 | 0.099/ 0.148 | 5.17E-05 | 0.64 | 0.119/0.123 | 0.573 | 0.96 | 0.515 | 0.115/0.129 | 0.013 | 0.88 [0.810.95] | 5.33E-03 |
| 7 | *CAV1* | rs2402091 | 0.095/ 0.142 | 5.34E-05 | 0.63 | 0.114/0.116 | 0.575 | 0.96 | 0.967 | 0.11/0.122 | 0.014 | 0.88 [0.80-0.97] | 0.02 |
| 7 | *-* | rs1228966 | 0.277/ 0.22 | 8.77E-05 | 1.37 | 0.208/0.205 | 0.521 | 1.02 | 0.072 | 0.222/0.208 | 0.015 | 1.09 [1.01-1.18] | 2.29E-03 |
| 7 | *SEMA3A* | rs1228870 | 0.275/ 0.218 | 7.62E-05 | 1.37 | 0.209/0.205 | 0.551 | 1.02 | 0.112 | 0.222/0.208 | 0.017 | 1.09 [ 1.01-1.18] | 3.15E-03 |
| 5 | *LOC389293* | rs7708428 | 0.379/ 0.45 | 4.54E-05 | 0.75 | 0.406/0.407 | 0.646 | 0.98 | 0.922 | 0.401/0.418 | 0.019 | 0.92 [0.87-0.98] | 0.01 |
| 9 | *XPA* | rs2808699 | 0.372/ 0.442 | 4.41E-05 | 0.75 | 0.411/0.416 | 0.620 | 0.98 | 0.020 | 0.403/0.423 | 0.021 | 0.92 [0.87-0.98] | 2.38E-04 |
| 8 | *DDEF1* | rs7839523 | 0.468/ 0.399 | 4.86E-05 | 1.32 | 0.433/0.434 | 0.647 | 1.01 | 0.099 | 0.44/0.425 | 0.021 | 1.07 [1.011-1.14] | 1.10E-03 |
| 9 | *XPA* | rs2805790 | 0.372/ 0.441 | 5.20E-05 | 0.75 | 0.410/0.415 | 0.611 | 0.98 | 0.025 | 0.403/0.422 | 0.022 | 0.92 [0.87-0.98] | 3.37E-04 |
| 3 | *IRAK2* | rs11706450 | 0.426/ 0.491 | 7.86E-05 | 0.75 | 0.474/0.479 | 0.548 | 0.97 | 0.262 | 0.465/0.482 | 0.024 | 0.93 [0.86-1.01] | 9.00E-03 |
| 9 | *XPA* | rs2805815 | 0.373/ 0.442 | 6.08E-05 | 0.76 | 0.411/0.416 | 0.642 | 0.98 | 0.023 | 0.403/0.422 | 0.024 | 0.93 [0.87-0.99] | 3.02E-04 |
| 2 | *NOL10* | rs4668690 | 0.097/ 0.061 | 8.05E-05 | 1.61 | 0.060/0.058 | 0.813 | 1.01 | 0.210 | 0.067/0.059 | 0.026 | 1.15 [1.01-1.30 | 3.10E-03 |
| 7 | *-* | rs1029541 | 0.288/ 0.227 | 2.37E-05 | 1.39 | 0.216/0.216 | 0.804 | 1.01 | 0.087 | 0.23/0.219 | 0.028 | 1.08 [1.01-1.17] | 8.07E-04 |
| 9 | *XPA* | rs2668797 | 0.37/ 0.441 | 2.90E-05 | 0.74 | 0.412/0.416 | 0.746 | 0.98 | 0.035 | 0.071/0.112 | 0.029 | 0.933 [0.87-0.99] | 2.48E-04 |
| 14 | *-* | rs1036570 | 0.277/ 0.342 | 4.88E-05 | 0.73 | 0.333/0.332 | 0.740 | 0.98 | 0.215 | 0.322/0.335 | 0.032 | 0.92 [0.85-1.014] | 2.13E-03 |
| 3 | *-* | rs4128236 | 0.347/ 0.284 | 6.60E-05 | 1.34 | 0.316/0.314 | 0.739 | 1.01 | 0.030 | 0.322/0.306 | 0.034 | 1.07[0.98-1.17] | 3.87E-04 |
| 1 | *-* | rs10925871 | 0.209/ 0.157 | 6.38E-05 | 1.41 | 0.188/0.189 | 0.790 | 1.01 | 0.427 | 0.193/0.181 | 0.038 | 1.08 [1.00-1.17] | 6.22E-03 |
| 7 | *CADPS2* | rs2501439 | 0.379/ 0.445 | 9.29E-05 | 0.76 | 0.427/0.428 | 0.744 | 0.98 | 0.385 | 0.418/0.432 | 0.042 | 0.93[0.87-0.99] | 7.49E-03 |
| 7 | *-* | rs757747 | 0.285/ 0.226 | 5.57E-05 | 1.37 | 0.215/0.215 | 0.925 | 1 | 0.113 | 0.229/0.218 | 0.047 | 1.07 [1.00-1.16] | 1.29E-03 |
| 7 | *WBSCR17* | rs4585627 | 0.353/ 0.288 | 3.42E-05 | 1.36 | 0.316/0.315 | 0.958 | 1 | 0.590 | 0.323/0.308 | 0.051 | 1.07 [ 1.00-1.14] | 4.70E-03 |
| 10 | *-* | rs1254860 | 0.138/ 0.098 | 9.69E-05 | 1.51 | 0.103/0.101 | 0.900 | 1 | 0.914 | 0.11/0.1 | 0.064 | 1.09 [0.99-1.21] | 0.03 |
| 8 | *DDEF1* | rs6470805 | 0.302/ 0.366 | 8.60E-05 | 0.75 | 0.340/0.336 | 0.993 | 0.99 | 0.423 | 0.333/0.344 | 0.069 | 0.94 [0.88-1.00] | 4.88E-03 |
| 9 | *LCN9* | rs541131 | 0.411/ 0.339 | 1.63E-05 | 1.36 | 0.397/0.401 | 0.834 | 0.99 | 0.805 | 0.4/0.385 | 0.071 | 1.06 [0.99-1.12] | 1.78E-03 |
| 5 | *CDH18* | rs2202798 | 0.096/ 0.059 | 2.60E-05 | 1.69 | 0.075/0.073 | 0.870 | 0.98 | 0.269 | 0.08/0.069 | 0.078 | 1.11 [0.98-1.24] | 9.17E-04 |
| 3 | *TDGF1* | rs6799581 | 0.244/ 0.307 | 5.35E-05 | 0.73 | 0.264/0.255 | 0.841 | 1 | 0.130 | 0.26/0.268 | 0.08 | 0.94 [0.86-1.03] | 6.85E-04 |
| 5 | *CDH18* | rs12655266 | 0.09/ 0.055 | 3.08E-05 | 1.71 | 0.070/0.068 | 0.737 | 0.97 | 0.347 | 0.074/0.065 | 0.111 | 1.10 [0.98-1.24] | 1.09E-03 |
| 4 | *NPY2R* | rs2880417 | 0.328/ 0.267 | 9.57E-05 | 1.33 | 0.283/0.286 | 0.806 | 0.99 | 0.003 | 0.292/0.281 | 0.117 | 1.05 [0.99-1.13] | 2.07E-05 |
| 7 | *-* | rs10272701 | 0.215/ 0.164 | 9.22E-05 | 1.39 | 0.186/0.189 | 0.769 | 0.98 | 0.821 | 0.192/0.183 | 0.13 | 1.06 [ 0.98-1.15] | 7.73E-03 |
| 8 | *FBX032* | rs3739284 | 0.203/ 0.261 | 9.31E-05 | 0.72 | 0.223/0.211 | 0.648 | 1.02 | 0.622 | 0.219/0.224 | 0.148 | 0.94 [0.87-1.01] | 3.71E-03 |
| 6 | *ASCC3* | rs7771570 | 0.536/ 0.463 | 1.82E-05 | 1.35 | 0.479/0.491 | 0.282 | 0.96 | 0.275 | 0.492/0.482 | 0.153 | 1.04 [ 0.98-1.11] | 2.97E-04 |
| 18 | *PHLPP* | rs2877745 | 0.103/ 0.066 | 3.40E-05 | 1.65 | 0.088/0.090 | 0.689 | 0.97 | 0.757 | 0.091/0.084 | 0.158 | 1.08 [0.98-1.18] | 3.49E-03 |
| 11 | *OPCML* | rs10894623 | 0.317/ 0.256 | 7.75E-05 | 1.34 | 0.285/0.289 | 0.615 | 0.98 | 0.257 | 0.291/0.281 | 0.175 | 1.04 [0.95-1.14] | 8.98E-04 |
| 21 | *-* | rs2831511 | 0.364/ 0.429 | 9.71E-05 | 0.76 | 0.400/0.394 | 0.651 | 1.01 | 0.026 | 0.393/0.403 | 0.175 | 0.95 [0.90-1.01] | 1.54E-04 |
| 6 | *ASCC3* | rs6919745 | 0.522/ 0.447 | 8.14E-06 | 1.37 | 0.464/0.474 | 0.414 | 0.97 | 0.248 | 0.476/0.467 | 0.19 | 1.042 [ 0.98-1.11] | 1.88E-04 |
| 4 | *NPY2R* | rs13138293 | 0.348/ 0.285 | 5.47E-05 | 1.34 | 0.298/0.304 | 0.528 | 0.97 | 0.027 | 0.308/0.299 | 0.195 | 1.05 [0.97-1.11] | 8.20E-05 |
| 15 | *SMAD3* | rs4147358 | 0.213/ 0.278 | 1.65E-05 | 0.7 | 0.243/0.231 | 0.380 | 1.03 | 0.565 | 0.237/0.243 | 0.203 | 0.95 [0.86-1.04] | 4.76E-04 |
| 17 | *TMEM132E* | rs4795032 | 0.384/ 0.315 | 2.93E-05 | 1.34 | 0.344/0.351 | 0.416 | 0.97 | 0.548 | 0.351/0.341 | 0.232 | 1.04 [0.95-1.13] | 6.65E-04 |
| 9 | *-* | rs10756265 | 0.288/ 0.354 | 4.80E-05 | 0.74 | 0.355/0.351 | 0.433 | 1.03 | 0.123 | 0.342/0.352 | 0.251 | 0.96 [0.90-1.02] | 2.18E-04 |
| 9 | *-* | rs443042 | 0.309/ 0.375 | 6.35E-05 | 0.75 | 0.380/0.376 | 0.444 | 1.02 | 0.084 | 0.366/0.376 | 0.261 | 0.96 [0.90-1.02] | 2.01E-04 |
| 9 | *SUSD3* | rs9696357 | 0.145/ 0.198 | 9.08E-05 | 0.69 | 0.156/0.147 | 0.344 | 1.04 | 0.721 | 0.154/0.16 | 0.268 | 0.95 [0.87-1.03] | 2.11E-03 |
| 12 | *SFRS8* | rs10794423 | 0.381/ 0.449 | 6.32E-05 | 0.75 | 0.453/0.440 | 0.401 | 1.03 | 0.501 | 0.439/0.442 | 0.282 | 0.96 [0.89-1.04] | 1.22E-03 |
| 2 | *ATP6V1C2* | rs7422405 | 0.406/ 0.475 | 4.55E-05 | 0.75 | 0.444/0.424 | 0.235 | 1.04 | 0.152 | 0.428/0.433 | 0.385 | 0.97 [0.91-1.03] | 1.93E-04 |
| 3 | *RBMS3* | rs35883 | 0.498/ 0.431 | 8.60E-05 | 1.31 | 0.447/0.465 | 0.170 | 0.95 | 0.097 | 0.457/0.456 | 0.556 | 1.019 [0.94-1.10] | 1.04E-04 |
| 21 | *CHODL \| PRSS7* | rs2248200 | 0.513/ 0.446 | 8.85E-05 | 1.31 | 0.485/0.492 | 0.775 | 0.98 | 0.458 | 0.484/0.481 | 0.598 | 1.01 [0.96-1.07] | 1.35E-03 |
| 21 | *CHODL \| PRSS7* | rs1688165 | 0.513/ 0.446 | 8.27E-05 | 1.31 | 0.488/0.493 | 0.894 | 0.99 | 0.324 | 0.485/0.482 | 0.628 | 1.01 [0.96-1.07] | 8.62E-04 |
| 16 | *ZNF423* | rs1477020 | 0.09/ 0.139 | 2.49E-05 | 0.61 | 0.128/0.118 | 0.095 | 1.09 | 0.070 | 0.121/0.123 | 0.649 | 0.97 [0.86-1.10] | 8.30E-06 |
| 1 | *C1QB* | rs631090 | 0.098/ 0.063 | 8.80E-05 | 1.61 | 0.067/0.072 | 0.089 | 0.88 | 0.570 | 0.073/0.070 | 0.729 | 1.02 [0.90-1.15] | 3.73E-04 |
| 16 | *ZNF423* | rs1990629 | 0.098/ 0.145 | 7.56E-05 | 0.64 | 0.135/0.125 | 0.100 | 1.09 | 0.067 | 0.128/0.130 | 0.736 | 0.98 [0.87-1.10] | 2.13E-05 |
| 11 | *OPCML* | rs11223273 | 0.302/ 0.244 | 9.91E-05 | 1.34 | 0.268/0.281 | 0.111 | 0.93 | 0.163 | 0.275/0.272 | 0.759 | 1.01 [0.92-1.10] | 1.40E-04 |
| 19 | *TSPAN16* | rs322151 | 0.285/ 0.227 | 7.79E-05 | 1.37 | 0.244/0.261 | 0.040 | 0.91 | 0.761 | 0.252/0.252 | 0.99 | 0.99 [0.94-1.06] | 3.37E-04 |

*Odds ratio for the minor allele

Chr, chromosome; CI, confidence interval; MAF, minor allele frequency; OR, odds ratio; *P*_BD_, Breslow–Day test *P*-value; *P*_MH_, allelic Mantel-Haenszel fixed effects model *P-*value; SNP, single nucleotide polymorphism.
