# Supplementary material for: A genome-wide association study follow-up suggests a possible role for PPARG in systemic sclerosis susceptibility
Source: Arthritis Res Ther. 2014 Jan 9;16(1):R6. doi: 10.1186/ar4432 (PMC3978735; doi:10.1186/ar4432)
Supplement: Additional file 2 — Genotype and minor allele frequencies of rs310746 and rs6832151 SNPs in two European cohorts (Replication-step). Description: this file contains: Additional file 2: Table S2 showing the genotype and allele distributions of rs310746 and rs6832151 genetic variants in two European cohorts (1032 SSc cases and 6700 controls). [file ar4432-S2.docx]

**Additional file 2: Table S2.** **Genotype and minor allele frequencies of rs310746 and rs6832151 SNPs in two European cohorts (Replication-step).**

|  |  |  |  |  | **Genotype, N (%)** | | |  | **Allele test** | |
| --- | --- | --- | --- | --- | --- | --- | --- | --- | --- | --- |
| **CHR** | **SNP** | ***Locus*** | **1/2** | **Subgroup (N)** | **1/1** | **1/2** | **2/2** | **MAF (%)** | ***P*-value** | **OR [CI 95%]*** |
| 3 | rs310746 | *SYN2\|PPARG* | C/T | **Italy** |  |  |  |  |  |  |
|  |  |  |  | Controls (n=1063) | 3 (0.28) | 133 (12.51) | 927 (87.21) | 6.54 |  |  |
|  |  |  |  | SSc (n=588) | 9 (1.53) | 82 (13.95) | 497 (84.52) | 8.50 | 0.037 | 1.33 [1.02-1.74] |
|  |  |  |  | **United Kingdom** |  |  |  |  |  |  |
|  |  |  |  | Controls (n=5632) | 69 (1.23) | 1096 (19.46) | 4467 (79.31) | 10.96 |  |  |
|  |  |  |  | SSc (n=444) | 11 (2.48) | 82 (18.47) | 351 (79.05) | 11.71 | 0.488 | 1.08 [0.87-1.33] |
| 4 | rs6832151 | *CHRNA9\|RHOH* | G/T | **Italy** |  |  |  |  |  |  |
|  |  |  |  | Controls (n=1048) | 100 (9.54) | 461 (43.99) | 487 (46.47) | 31.54 |  |  |
|  |  |  |  | SSc (n=580) | 60 (10.34) | 244 (42.07) | 276 (47.59) | 31.38 | 0.926 | 0.99 [0.85-1.16] |
|  |  |  |  | **United Kingdom** |  |  |  |  |  |  |
|  |  |  |  | Controls (n=5637) | 409 (7.26) | 2264 (40.16) | 2964 (52.58) | 27.34 |  |  |
|  |  |  |  | SSc (n=442) | 31 (7.01) | 180 (40.72) | 231 (52.26) | 27.38 | 0.980 | 1.00 [0.86-1.17] |

*Odds ratio for the minor allele. Chr, chromosome; CI, confidence interval; MAF, minor allele frequency; OR, odds ratio; SNP, single nucleotide polymorphism; SSc, systemic sclerosis.
